# Species-Specific Duplication Event Associated with Elevated Levels of Nonstructural Carbohydrates in *Sorghum bicolor*

**DOI:** 10.1534/g3.119.400921

**Published:** 2020-03-04

**Authors:** Zachary W. Brenton, Brendon T. Juengst, Elizabeth A. Cooper, Matthew T. Myers, Kathleen E. Jordan, Savanah M. Dale, Jeffrey C. Glaubitz, Xiaoyun Wang, Richard E. Boyles, Erin L. Connolly, Stephen Kresovich

**Affiliations:** * Advanced Plant Technology, Clemson University, Clemson, SC; † Department of Plant and Environmental Sciences, Clemson University, Clemson, SC; ‡ Department of Plant Science, Penn State University, State College; § Department of Bioinformatics and Genomics, University of North Carolina at Charlotte, Kannapolis, NC; ** Institute for Biotechnology, Cornell University, Ithaca, NY

**Keywords:** Neofunctionalization, Duplication, Carbon partitioning, Poaceae, Adaptation

## Abstract

Simple sugars are the essential foundation to plant life, and thus, their production, utilization, and storage are highly regulated processes with many complex genetic controls. Despite their importance, many of the genetic and biochemical mechanisms remain unknown or uncharacterized. Sorghum, a highly productive, diverse C_4_ grass important for both industrial and subsistence agricultural systems, has considerable phenotypic diversity in the accumulation of nonstructural sugars in the stem. We use this crop species to examine the genetic controls of high levels of sugar accumulation, identify genetic mechanisms for the accumulation of nonstructural sugars, and link carbon allocation with iron transport. We identify a species-specific tandem duplication event controlling sugar accumulation using genome-wide association analysis, characterize multiple allelic variants causing increased sugar content, and provide further evidence of a putative neofunctionalization event conferring adaptability in *Sorghum bicolor*. Comparative genomics indicate that this event is unique to sorghum which may further elucidate evolutionary mechanisms for adaptation and divergence within the Poaceae. Furthermore, the identification and characterization of this event was only possible with the continued advancement and improvement of the reference genome. The characterization of this region and the process in which it was discovered serve as a reminder that any reference genome is imperfect and is in need of continual improvement.

The continued existence of biology on Earth is largely dependent on the creation of sugar generated through carbon fixation. As one of the most fundamental sources of energy for life, understanding the biological controls of the synthesis and regulation of sugar flux in plants is critical for the continued improvement of agricultural productivity regardless of system. Sugar is not only used as a source of energy for plant cells, but it is also used as a signaling molecule for key developmental and physiological changes spanning the life cycle of the plant. Sugar and its myriad biological regulators and sensors are linked to seed development and growth, hormone signaling, carbon metabolism, stress response and programmed cell-death initiation (Rolland *et al.* 2006). Despite the essential nature of simple sugars in plant biology, many of the biological pathways, signals, and genetic and biochemical mechanisms remain unknown, uncharacterized, or, at best, incomplete (Smeekens and Hellmann 2014).

Due to their ecological and economic importance and phenotypic diversity, the Andropogoneae, a clade within the Poaceae that includes *Zea mays*, *Saccharum officinarum*, *Panicum virgatum* and *Sorghum bicolor*, serves as an excellent and relevant system for understanding the genetic determinants of sugar accumulation. Not only are these plants critical in the food production system, but some (*i.e.*, sugar cane and sweet sorghum) are also largely grown for their harvestable supply of nonstructural sugars. Sorghum, a C_4_ grass native to the Horn of Africa, is a diverse species (Mace *et al.* 2013b; Lasky *et al.* 2015) with a complex history; this diversity has allowed for its utilization in both subsistence and industrial agricultural systems as a source of food, feed, fuel and fiber (Paterson *et al.* 2009). High levels of sugar accumulation may reduce sensitivity to salinity (Ashraf and Harris 2004) and drought (Premachandra and Hahn 1995). Accumulation of sugars is also believed to play a pivotal role in disease resistance (Bolouri Moghaddam and Van den Ende 2012). Due to the economic and ecological relevance of sugar accumulation in sorghum, previous work has characterized the physiological controls and inheritance patterns of sugar accumulation (Lingle 1987; Hoffmann-Thoma *et al.* 1996; Murray *et al.* 2008b 2009; Tarpley *et al.* 1994), which in turn has led to genetic studies (Bihmidine *et al.* 2016; Calviño and Messing 2012), expression analysis (McKinley *et al.* 2016; Cooper *et al.* 2019), and a concerted breeding effort (Kresovich *et al.* 1988; Rooney *et al.* 2007); however, the specific genetic controls of increased sugar accumulation are largely unknown and uncharacterized.

We analyzed the entire stalk for water-soluble sugars, which is where the majority of these sugars accumulate, with a genome-wide analysis study (GWAS) using a diversity panel that was specifically designed to dissect the genetic determinants of biomass constituents (Brenton *et al.* 2016; Boyles *et al.* 2018). The GWAS identified four regions associated with sugar accumulation, and the genes linked to these associated SNPs did not have any functional annotation that would imply any effect on sugar accumulation. Comparison between the two sorghum reference genomes, one chosen for its high levels of sugar accumulation, allowed us to specify potential candidate genes for additional sequencing. Additional sequencing revealed a relationship between certain alleles of a tandem duplication of vacuolar iron transporters on Chromosome 4. A heterologous complementation study demonstrated that the alleles from individual genotypes with high levels of sugar accumulation restored functionality in a yeast mutant that lacks a functional vacuolar iron transporter. Further evidence suggests that the phenotype is not caused by increased dosage or a functional-to-non-functional dichotomy, but rather the data imply that this is a neofunctionalization event and the copies vary in localization patterns. Despite the suggestive evidence, this hypothesis has yet to be fully vetted.

More importantly, this study serves as a reflection on the relationship of genomic analysis with the quality of the reference genome. These findings would not have been possible without the creation of the second sorghum reference and the improvement underlying assembly of the first reference genome. Sorghum, which was the 4^th^ plant genome to have a full reference sequence, is considered a ‘gold-standard’ reference since it was originally assembled using Sanger sequencing as opposed to next generation sequencing (VanBuren *et al.* 2015). Considering the relative quality of the sorghum reference, it is surprising to find a gene duplication event was missing from any analysis nearly a decade after the first published genome, and then later associate this duplication with an adaptive trait in subsequent analysis. This study can serve as an example and justification for the scientific community to continue to invest in the creation and dissemination of improved reference genome assemblies rather than sequencing more individuals at lower coverages.

## Materials and methods

### Germplasm, field design, and phenotyping

Phenotypic data collection was conducted on the Sorghum Biomass Association Panel (BAP) (Brenton *et al.* 2016), a previously defined diversity panel with 390 accessions, at the Clemson University Pee Dee Research and Education Center in Florence, South Carolina in 2014 and 2015. Each year two replicates of the BAP were planted on 76cm rows in a complete randomized block design at approximately 96,000 plants/ha. Seed for the BAP was obtained through the United States Department of Agriculture’s Germplasm Resources Information Network. Prior to planting, seed was treated with a chemical slurry of Concep II, Nipsit, Apron XL, and Maxim XL. This enabled the spraying of Bicep II Magnum, a combination of Atrazine and S-Metolachlor, prior to seed germination at a rate of 3.5 L ha^-1^ for control of grasses and broadleaf weeds. At approximately 35 days after planting, a second application of Atrazine was applied at a rate of 4.7 L ha^-1^ tanked mixed with 125 kg ha^-1^ of layby nitrogen. In 2014, no other fungicides or insecticides were applied. In 2015, the insecticide Transform was applied at a rate of 110 ml ha^-1^ to control sugarcane aphid. In both years, the BAP was planted in fields with pivot irrigation. Irrigation was applied at planting. At about 90 days, the plants had reached a height that prevented irrigation with the pivot system. The selected individuals for candidate gene sequencing were phenotyped in 2017 at the Clemson University Simpson Farm Research Station in Pendleton, South Carolina under similar management conditions.

Samples for compositional analysis were harvested at either physiological maturity, or an October 1^st^ cutoff for photoperiod sensitive accessions. Samples consisted of three representative plants per plot that were dried with a forced-air heated dryer at approximately 40C. Once samples had reached a constant weight, samples were ground to a 2mm particle size using a Wiley Mill. The compositional phenotypes cellulose, hemicellulose, lignin, and water-soluble carbohydrates were measured using near-infrared spectroscopy (NIRS) on a Perten DA7250. Cellulose and hemicellulose were estimated based on acid detergent fiber (ADF) and neutral detergent fiber (NDF) (Murray *et al.* 2008a). Protocols for lignin, ADF, and NDF compositional analysis were previously described (Brenton *et al.* 2016). The NIR curve for Water-soluble carbohydrates (WSC) was developed with collaboration from the Perten Applications team with analysis from Dairyland Labs (www.dairylandlabs.net) using a previously described protocol for water-soluble carbohydrates (Deriaz 1961). Data for curve calibrations are available in File S1. Yield was the total dry weight of the three harvested plants. Compositional data were based on the stems and tillers; leaves and panicles (if present) were stripped before grinding. Phenotypic values are available in File S2.

### Correlations and heritability

**Figure 1:**
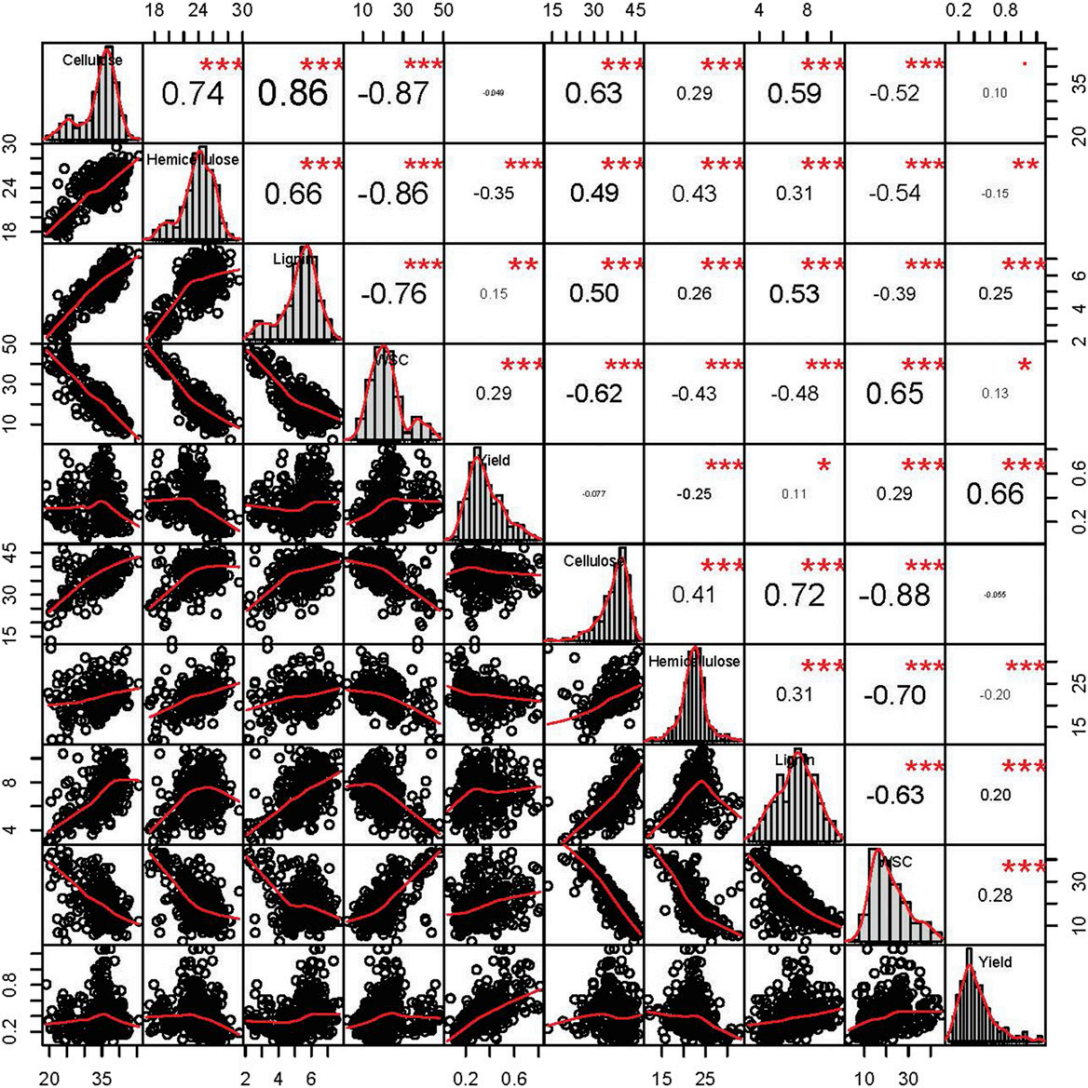
Using Pearson Correlation, the correlations and distributions for both years of phenotypic data are shown for cellulose, hemicellulose, lignin, water-soluble carbohydrates (WSC), and yield. Compositional data are presented as the percent of dry matter, and yield is presented in kg.

Heritability and variance component calculations were made using the R Package ‘Heritability’ (Kruijer *et al.* 2015). The marker-assisted heritability measurements ($h^{2}$) were calculated with a centered relatedness matrix from the software GEMMA (Zhou and Stephens 2012). The centered relatedness matrix utilized the same SNPs as the GWAS. Correlations were calculated using a Pearson correlation method. The R Package ‘Performance Analytics’ enabled the creation of Figure 1. All of the phenotypic values are expressed as percent of dry matter.

### SNP calling, genome-wide association analysis, and candidate gene identification

As previously described (Brenton *et al.* 2016), the genomic data for the BAP was generated through genotyping-by-sequencing (Elshire *et al.* 2011) libraries using ApeKI enzymatic digestion. Sequencing was performed on an Illumina HiSeq 2000. The single-end reads for the BAP have been deposited in the NCBI SRA under BioProject identification number PRJNA298892. Single nucleotide polymorphisms were called using the TASSEL 5.0 pipeline (Bradbury *et al.* 2007) and Burrows-Wheeler alignment (Li and Durbin 2009). A minimum aligned read depth of 10 was required for calling SNPs in an individual. Sequence data were mapped back to the sorghum reference genome (Paterson *et al.* 2009) version three available on Phytozome (Goodstein *et al.* 2012). Missing genotypic information was imputed to the 80% confidence threshold using the software fastPHASE (Scheet and Stephens 2006).

The genome-wide association study was performed using version two of the R Package ‘GAPIT’ (Lipka *et al.* 2012; Tang *et al.* 2016). Before association scans were performed, SNPs were filtered by a minimum allele frequency of 5% and present in at least 165 individuals (approximately 50% of the individuals with phenotypic data). Within the GAPIT package, the association scans utilized a compressed mixed linear model (CMLM) which internally calculates and adjusts for kinship (K) and population structure (commonly referred to as a Q matrix). The incorporation of both kinship and population structure in GWAS reduces the occurrence of Type I errors. To further limit the possibility of false positives, an extremely conservative threshold, the Bonferroni correction method, was used to determine significance of associated SNPs. For a SNP to be considered significant using the Bonferroni correction method, the p-value had to be less than 2.83 x $10^{-7}$. GWAS was conducted on 334 individuals using 176,380 SNPs. Phenotypic values for GWAS were calculated using best linear unbiased prediction using the R package ‘lme4’ (Bates *et al.* 2014) from both replicates from 2014 and 2015. All effects were treated as random.

Multiple studies have estimated genome-wide linkage disequilibrium (LD) decay in sorghum ranging from 15-20 kb (Hamblin *et al.* 2004; Mace *et al.* 2013b) up to 150kb (Morris *et al.* 2013). Since LD can vary significantly across the genome (Mace *et al.* 2013b), we calculated LD locally within 1 MB of significantly associated SNPs using the R Package ’Genetics’ for every independent associated genomic region. LD was considered to decay when the $r^{2}$ was less than 0.1 (File S3). Any gene that was within the local LD estimates of a significantly associated SNP was considered potentially causative and reported as a putative candidate gene. All gene information was obtained through Phytozome along with information for cross-species comparisons (Goodstein *et al.* 2012). The program SNPeff was used to annotate SNP function (Cingolani *et al.* 2012).

### Phylogenetic analysis and evolutionary comparisons

Phylogenetic comparisons consisted of the amino acid sequences from sorghum to the other putative vacuolar iron transporters (VITs) within the Poaceae. Phylip’s protein parsimony algorithm was used to find the best tree given the amino acid sequences from Phytozome. The tree was constructed using the Interactive Tree of Life software (Letunic and Bork 2016). Comparisons among the species were made using Phytozome’s Gene Ancestry information. Figure 3 is a rendition of the Cluster Identifier 73757671 on Phytozome.

### Candidate gene sequencing

A total of 17 individuals were sequenced: 16 diverse accessions from the BAP and BTx623, the reference genome, as a control. The 16 individuals were selected based on the segregation of SNPs S4_64019119 and S4_64019913, historical importance in sweet sorghum breeding, variation in composition of nonstructural sugars, and a limited range in anthesis date to remove any confounding variables. The Cornell University Institute of Biotechnology’s Genomic Facility extracted the DNA, developed primers, and sequenced Sobic.004G301500. DNA was extracted directly from seed using the Qiagen Plant DNA extraction kit. Primers were developed using the reference sequence (BTx623) on Phytozome. Additional seed of BTx623 was sent as a control. Sanger sequencing was performed using the left and right primer sequences GGATTCATTCTAGGTATCGAACGAGC and GCCAATTGTGATCCATTTATTGCCAG, respectively. The final sequencing product included the entire coding region of the gene, the promoter region, and approximately 100bps upstream and downstream of the target gene. Gene sequences were aligned using Clustal W alignment (Thompson *et al.* 1994) available through the EMBOSS suite of bioinformatic programs (Chojnacki *et al.* 2017). The haplotype network (Figure 4) was created using a R script that includes R packages ‘Ape’ (Paradis *et al.* 2004) and ‘Pegas’ (Paradis 2010). Aligned sequences are available in File S4.

### Yeast complementation

The two sorghum alleles were synthesized and confirmed by Genscript based on the open reading frame sequences from Rio and BTx623. These alleles were PCR amplified using the same forward primer R/BF 5′ ATGCATGGTACCATGTTTTACGCCTTTGCTGATAAGC 3′ and separate reverse primers RioR 5′ ATGCATCTCGAGGATGCGGACGACAAGACATGC 3′ and BTx623R 5′ ATGCATCTCGAGGATGCGGACGCGACA TGCGTC 3′ and then cloned into gateway entry vector pENTR4, before being transferred to the Gateway yeast destination vector pAG426-GAL-EGFP. Saccharomyces cerevisiae strain DY150Δccc1 was transformed with pAG426-GAL-EGFP or pAG426 carrying either the Rio allele, BTx623 allele, or AtVIT1 (positive control). The yeast *ccc1* knockout and the AtVIT1 construct were obtained from Professor Mary Lou Guerinot at Dartmouth College. The experiment was modeled after previously reported methods from her lab (Kim *et al.* 2006). For the complementation assay SD-ura plates supplemented with 2% galactose and either no added iron, 5mM FeSO_4_ or 8mM FeSO_4_ were used. The yeast strains were cultured for 2 days in SD-ura liquid supplemented with 2% Raffinose, then washed and diluted to an OD_600_ of 1.0 with sterile distilled water. Yeast cells were then plated in 10µL aliquots on 8mM FeSO_4_ plates or diluted 10-fold and plated on 5mM FeSO_4_ plates and grown for 5 days at 30C. We performed qPCR to confirm that all constructs were successfully expressed in yeast (File S5).

### Data availability

All germplasm used in this study is publicly available through the USDA-ARS Germplasm Repository Information Network. Genomic data are available through PRJNA298892. File S1 explains development of NIR curves. File S2 provides the phenotypic data used in the analysis. File S3 provides the graphs of LD decay. File S4 is the alignment files for candidate genes. File S5 is a more thorough explanation of yeast complementation study. File S6 is the GWAS results and the full list of genes within LD. File S7 is the multiple sequence alignment file for the candidate genes. File S8 lists the phenotypic values and correlations used to make Figure 4. File S9 is the visual representation of the changes to the sorghum reference genome over time. Supplemental material available at figshare: https://doi.org/10.25387/g3.11929392.

## Results

### Trait correlations and heritability

WSC were negatively correlated with cellulose, hemicellulose, and lignin as a percentage of dry matter (Figure 1). Individual components were positively correlated with each other year over year which also corresponds to the relatively high heritability. The heritability year over year and within years demonstrates that WSC is clearly genetically influenced and amenable to selection. The heritability of WSC is generally as high as if not higher than Brix measurements (Murray *et al.* 2009), which measures the solutes of aqueous solutions. Although BRIX is prevalently used to estimate sugar content in sweet sorghum, the higher heritability of WSC may suggest that this methodology is superior for correctly determining sugar content in sorghum in phenotypically diverse panels. Overall, the heritability estimates are fairly consistent within and across years (Table 1).

**Table 1:** Heritability of WSC

	$H^{2}$	$h^{2}$	$V_{G}$	$V_{A}$	$V_{E}$
2014	0.70	0.71	56%	57.6%	23.0%
2015	0.65	0.60	52.8%	43.5%	28.8%
Both years	0.59	0.56	47.9%	42.2%	32.7%

Narrow and broad sense heritability for replicates per year and year over year. $V_{G}$ is variance explained by genetics, $V_{A}$ is additive genetic variance, and $V_{E}$ attributed to the environment.

Interestingly, WSC has the highest correlation with yield of any of the biomass components. It is known that lignin, a key structural component, is correlated with increased biomass (Pedersen *et al.* 2005). Multiple studies in forage sorghum and other plant species demonstrate that lignin mutants reduce overall biomass yield (Sattler *et al.* 2010). Given the known relationship between lignin and yield, it is surprising that sugar content would have a much stronger correlation with yield than lignin. Previous studies have suggested that sugar content can impact sorghum’s tolerance of biotic and abiotic stress, but since the individuals in this study were not exposed to heavy drought, saline conditions, or agents of disease, this data indicates that sugar may play a more crucial role in final plant yield. Neither anthesis nor harvest date were highly correlated with WSC (File S2). The weak correlation is due to the design of the panel, which was specifically constructed to minimize the confounding effects of height and maturity (Brenton *et al.* 2016).

### GWAS of WSC

**Figure 2:**
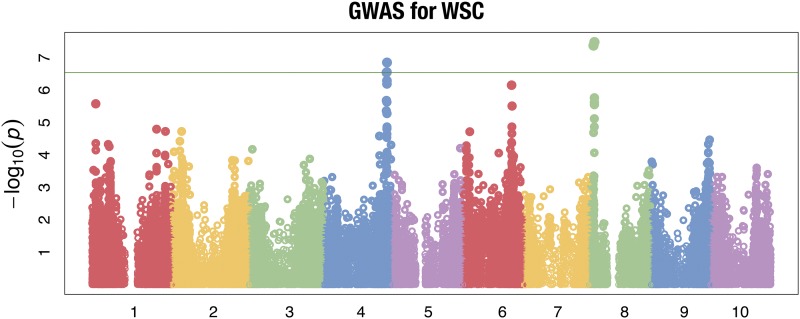
The x-axis corresponds to the genomic position of each SNP represented by a dot on the graph. The y-axis is the -log p-value of each SNP. Each color corresponds to a chromosome. The red horizontal line is the statistical threshold used in this study, the Bonferroni correction.

Using the compressed mixed linear model (CMLM) from GAPIT 2, the GWAS identified a total of five SNPs that passed the Bonferroni significance threshold (Table 2). These five SNPs correspond to four independent regions (two on Chromosome 4 and two on Chromosome 8)GWAS. Based on the local LD calculations for each region (File S3), a total of 14 genes appear to be linked to associated SNPs (File S6). Furthermore, these results corroborate earlier studies that identified the same locus on Chromosome 4 for NFC (Brenton *et al.* 2016), a less specific measurement of nonstructural components; now that the GWAS for WSC has narrowed possible candidates to Chromosome 4, it strongly suggests that the loci on Chromosome 4 impact nonstructural sugar accumulation in sorghum (Figure 2).

**Table 2:** Significant SNPS identified through GWAS

SNP position	P-value	Minor allele frequency	Major/minor allele	Local LD (kb)	Number of genes within LD	Allelic effect
S4_63511857	2.83 x 10${}^{-7}$	11.0%	C/G	17	5	3.68
S4_64019119	2.72 x 10${}^{-7}$	9.1%	A/C	23	6	4.36
S4_64019913	1.39 x 10${}^{-7}$	9.1%	A/C	23	6	−4.55
S8_4595588	4.19 x 10${}^{-8}$	11.3%	A/G	7	2	3.55
S8_5592568	3.25 x 10${}^{-8}$	8.1%	C/G	6	1	4.57

Results from WSC GWAS: significantly associated SNPs, allele frequency, locally calculated LD, number of genes within LD, and the estimated allelic effect of on WSC.

Although numerous studies have attempted to explicate the genetic basis of increased nonstructural sugars in sorghum, causal genetic variants have yet to be identified. Many of these studies have looked at expression of known genes such as sugar transporters and invertase enzymes (Milne *et al.* 2013) rather than utilizing a genome-wide approach to explore natural variation and experimentally implicate possible causal loci. Since none of the candidate genes identified through GWAS have known functions relating to sugar accumulation, transport, or metabolism, perhaps the accumulation of high levels sugar in sorghum is due to genes with uncharacterized functionality. The accumulation of nonstructural sugars may also be due to a sorghum-specific genetic variant, which is why homology driven approaches based on *a priori* candidates may not have yielded the results researchers intended, and that genome-wide analysis may provide more clarity on which genetic determinants increase sugar accumulation.

#### Eliminating false positives:

Diversity panels have a propensity for higher occurrences in false positives in large part due to physiological and timing differences. The two most obvious phenotypic variables that could confound genotypic-phenotypic associations for accumulation of sugar in sorghum are flowering time (*i.e.*, anthesis) and height. Independent GWAS results for both height and flowering time did not reveal any loci that co-localized among phenotypes. Although recent evidence has disproven the relationship of sugar content and height (Shukla, S. and Felderhoff, T. and Saballos, A. and Vermerris, W. 2017), both the known height and maturity genes (Mace and Jordan 2010) were compared against the GWAS results for co-localization; the regions associated with height and flowering time do not correspond to any of the genomic loci identified through GWAS of WSC. Furthermore, none of the candidate genes associated with WSC correspond to the nearly 200 candidate genes identified in previous genomic studies used to dissect flowering time in sorghum (Mace *et al.* 2013a).

### Candidate genes linked to associated SNPs

The results from this study did not overlap with previously identified regions identified by either QTL or association analysis (Murray *et al.* 2008b, a; Burks *et al.* 2015). The associated regions did not display genes that were significantly differentially expressed between grain and sweet types (McKinley *et al.* 2016; Cooper *et al.* 2019). The genetic studies may not have overlapped due to the differences in phenotyping methodologies; previous studies used BRIX to estimate sugar content whereas this study utilized whole plant NIR. BRIX measurements are a function of sugar content and moisture which is why BRIX readings were able to successfully identify the *Dry midrib (D)* allele in sorghum (Burks *et al.* 2015), but may not have identified the drivers of increased sugar accumulation. Since the literature did not seem to corroborate the associated genomic regions, genomic sequence was compared between the original reference genome (BTx623) and the Rio assembly. BTx623 is a critically important grain sorghum breeding that accumulates low to moderate amounts of nonstructural sugars whereas Rio is a historically important sweet sorghum breeding line that accumulates high levels of nonstructural sugars in the stalk. Genomic and amino acid sequences within the identified region on Chromosome 4 (SNPs S4_64019119 and S4_64019913) were compared between BTx623, the first reference genome, and Rio, the *de novo* assembled archetypal sweet sorghum reference genome (Cooper *et al.* 2019) to determine possible candidate genes.

**Figure 3:**
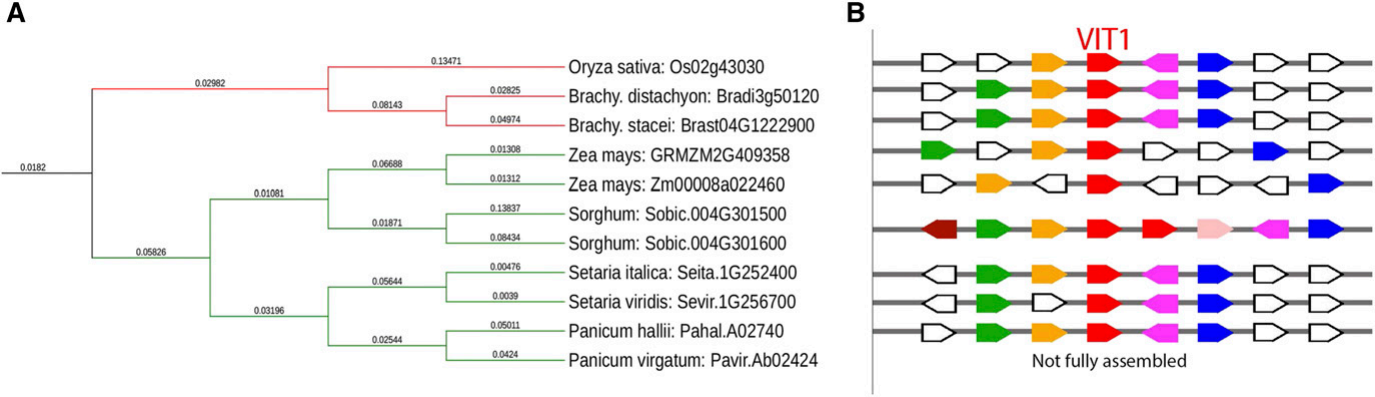
A) Phylogenetic tree construction of the peptide sequence of VIT1 from the Poaceae. The red line indicates C_3_ species and the green line indicates C_4_. Both sequences and gene IDs were obtained through Phytozome. The tree was using Tree of Life software. B) Genomic synteny viewer of the grasses. The red figures indicate high homology with VIT1. Like colors indicate putatively shared functions. Plain white figures indicate a gene with no shared homology to sorghum. Each line matches the species in part A. Sorghum has a duplication unique to the grasses (sobic.004G301500 and sobic.004G301600). The shaded pink (sobic.004G301650) indicates a possible additional VIT1. However, there is no expression data and given its truncated nature it has a high possibility of being a pseudogene.

**Figure 4:**
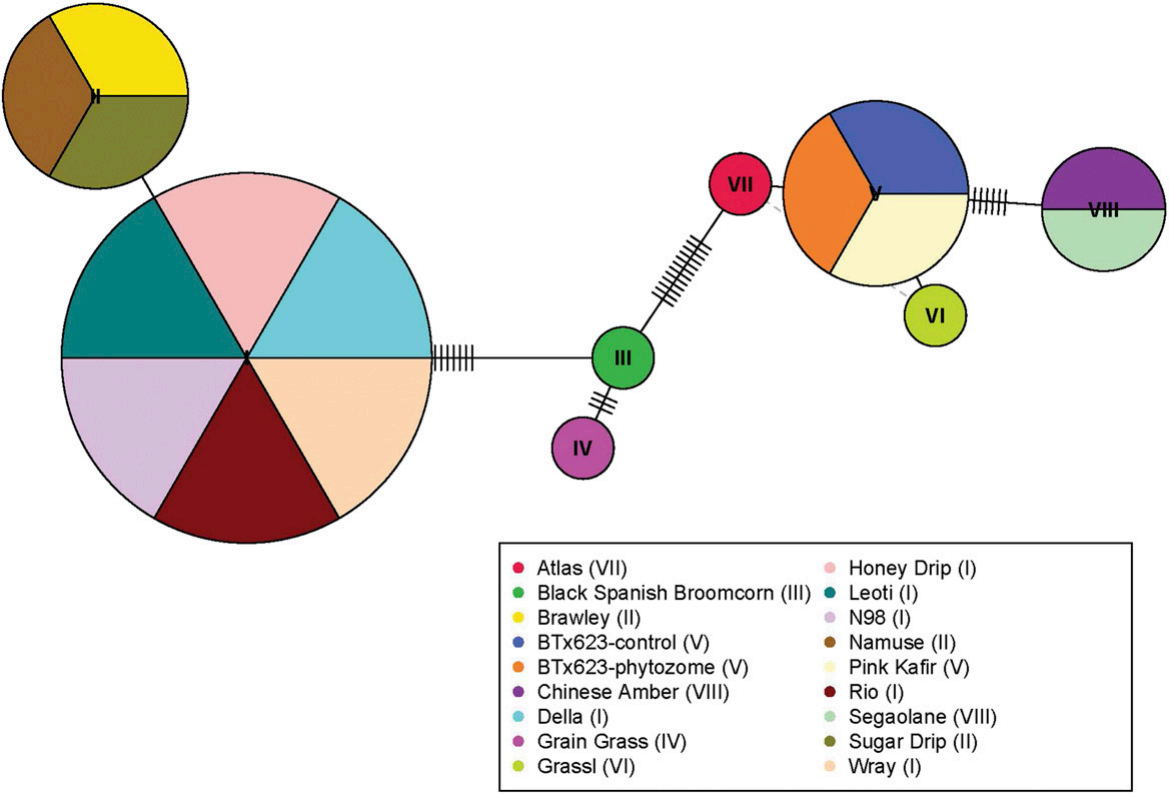
Allelic network of the 16 individuals, the control BTx623, and the reference sequence on Phytozome. Values reflect the average WSC as a percentage of dry matter of two groups: alleles I and II and the remaining six alleles. Raw phenotypic values for each individual accession along with values for each individual allele can be found in File S8.

The region identified through GWAS on Chromosome 4 contains a novel sorghum duplication (Figure 3), a series of nodulin-like 21 genes which share a low homology to AT3G43660, a putative vacuolar iron transporter (VIT) (Gollhofer *et al.* 2011). The nodulin-like21 family is a diverse collection of integral membrane proteins whose functions range from the transport of sugar to amino acids to auxin (Denancé *et al.* 2014). Originally named for their role in symbiosis in leguminous species, the nodulin-like families of genes still are largely uncharacterized in non-nodulating species like the grasses. Based on the sequence variation of these alleles between the grain and sweet reference genomes and the evolutionary hypotheses of the role of gene duplications in plant adaptation (Flagel and Wendel 2009), the duplication event became the most likely cause of increased sugar accumulation. There were no significant differences in the sequences of Sobic.004G301600 between Rio and BTx623, so it was eliminated as the causal gene. Expression data are available in multiple tissues at multiple timepoints for both Rio and BTx623, which is why sequence comparisons were the primary determinant of a gene’s estimated effect. Since Sobic.004G301500 diverged considerably between the two references (File S7), this gene was identified as a putative candidate worthy of additional efforts to demonstrate a causal relationship with WSC. Amplicon sequencing was performed on Sobic.004G301500. The selected individuals for sequencing were grown again in a contrasting environment in 2017 to see if similar patterns of sugar accumulation remained. Figure 4 shows that individuals with alleles I and II accumulate high levels of sugars (22%) whereas individuals with other alleles accumulate low to moderate levels of sugar (13%).

A phylogeny was constructed based on amino acid sequences from the two possible duplicates and the closest relatives of sorghum within the Poaceae. The phylogeny clearly demonstrates that the sorghum copies cluster together, suggesting that our candidate gene is a novel copy that arose after sorghum diverged from the most recent common ancestor. We hypothesize that it is more likely that an expansion occurred in this region after divergence rather than all of the other Poaceae losing a copy of this gene. Despite their shared ancestry, the publicly available expression data available through Phytozome shows divergent expression patterns between these two genes. Mutations within these two genes are outside of the *ccc1* domain which is the responsible for iron transport. This suggests that these genes have either developed totally different functions in sorghum or different localization patterns, which would qualify this duplication as a potential neofunctionalization event. Transcriptomics data highlights the divergent patterns of transcription in various tissues at varied development time points (Shakoor *et al.* 2014; Makita *et al.* 2015) between the genes. This would suggest that one of these genes developed a new function, possibly one affecting patterns of sugar accumulation. If this is not the case and if each gene still retains the function of a VIT, then it is possible that these genes regulate sugar accumulation via an iron deficiency response as was shown in *Arabidposis* (Lin *et al.* 2015).

In addition to the high levels of sugar content, the individuals with Alleles I and II have an approximately 18% yield increase over the six alleles. Furthermore, the positive correlation between yield and sugar content was much stronger in this small subset than the broader BAP (File S8). This is surprising because individuals were not selected for additional sequencing based on yield. They were selected on their segregation of the associated alleles, the observed sugar content, and the historical value in both syrup and sugar breeding programs. This unexpected result lends credence to the claim that sugar may be a key phenotype in driving increased biomass yield, rather than just a tradeoff between structural and nonstructural compositional components.

#### Molecular characterization of duplicated genes:

**Figure 5:**
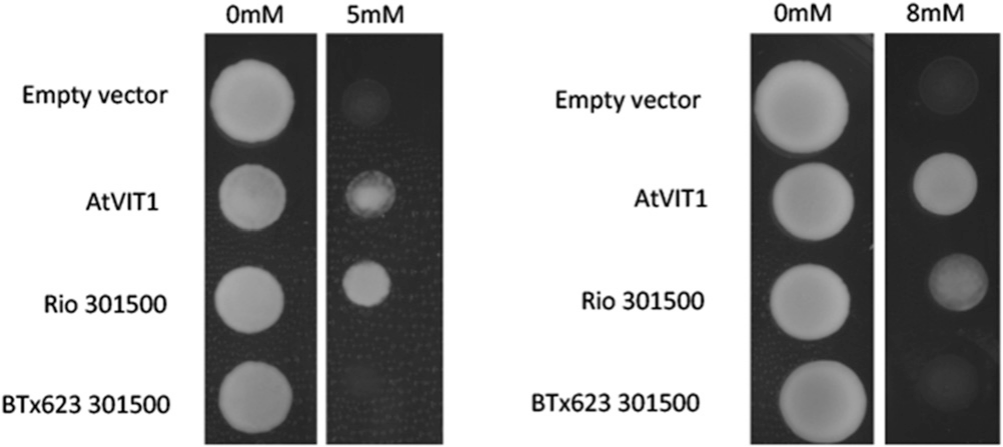
Tests of functional complementation of the *ccc1* yeast strain with VIT1 alleles. The iron sensitive Δccc1 yeast strain was transformed with either an empty vector, Arabidopsis VIT1 (positive control) or the two Sorghum alleles and the resulting strains were grown on Synthetic Defined media supplemented with no iron (control), 5mM or 8mM FeSO_4_. Yeast strains were spotted in 10µL volumes at OD_600_ values of 1.0 for 8mM and 0.1 for 5mM. *AtVIT* and the Rio allele rescue the iron sensitivity phenotype of *ccc1* while the BTx623 allele does not.

Since the predicted function of Sobic.004G301500 is vacuolar iron transport, we decided to use sorghum alleles from the two sorghum reference genomes, Rio and BTx623, to express in yeast lacking the vacuolar iron transporter *ccc1* (Ca^2+^ Calcium Cross Complementer 1). Loss of *ccc1* causes an inability to store iron in the vacuole which leads to buildup of iron in the cytoplasm, and an iron hypersensitivity phenotype (Li *et al.* 2001). The Rio allele and the Arabidopsis Vacuolar Iron Transporter 1 (positive control) rescued growth of *ccc1* suggesting they can transport iron into the vacuole whereas the BTx623 allele did not (Figure 5). Since the predicted *ccc1* domain was conserved among the two sorghum alleles and the Arabidopsis VIT1, it was not expected that the two alleles would display differing abilities to rescue the Fe hypersensitivity. qPCR confirmed that both constructs were expressed in the yeast. Since the major differences in the two alleles are unknown functional domains, it is possible that this putative neofunctionalization event is due to changes in localization patterns.

## Discussion

### The importance of improved genomic resources

The sorghum reference genome is often considered a ‘gold standard’ since it was completely assembled from Sanger sequencing rather than from next-generation short read sequences (VanBuren *et al.* 2015). Newer genome versions were able to build upon and enhance an already strong foundation. However, the identification of this putative neofunctionalization event would not have been possible without the continued improvement of the reference genome. In each version of the genome, an additional copy of VIT was identified (File S9). The revelations surrounding the expansion in this region along with the experimentally implicated GWAS results may simultaneously serve both positive and negative exemplary roles; on one hand, this characterization further validates the use of long-read SMRT sequencing technology, and on the other, raises serious questions about potential missing data in other plant genomes.

### Determining the drivers of improved productivity

Cropping systems must become more productive in light of unpredictable climatic changes; in order to do so, the crops themselves must become more resilient in the face of these challenges. However, the genetic determinants of crop productivity remain poorly understood. In terms of source/sink dynamics, much emphasis is placed on improving source sequestration (*i.e.*, photosynthetic activity) with the underlying assumption that increasing available photosynthate translates to improved yield. This assumes that the limiting factor of plant growth is indeed photosynthetic efficiency and that both plant metabolic processes and carbon-sink relationships are fully optimized. The data presented here suggests that increasing available sugars actually increases overall plant yield and sink strength rather than the simple reallocation of carbon among various sinks; if the relationship between increased productivity and increased sugar content is true, it would indicate that the genetic determinants influencing carbon allocation are not optimized and that greater productivity gains could be achieved by focusing on improved sink strength.

GWAS remains an extremely powerful method for identifying genomic variants controlling phenotypic variation. The identification of SNPs that overlap with a novel duplication event unique to sorghum not only serves as an intriguing target for crop improvement, but also supports the hypothesis that duplication events are critical for adaptation and improved fitness by developing novel functionality and increasing phenotypic variation. Since duplicated genes can gain a new function or be relegated to oblivion over time through evolutionary redundancy, the retention of this duplication event in sorghum offers promise that this region may be of importance not only for the elucidating the evolution of sorghum, but also may be important for continuing crop improvement efforts. Despite strong evidence for a possible neofunctionlization event, molecular techniques, specifically transgenic methodologies, will need to be employed for definitive confirmation. To test this hypothesis, we recommend editing Sobic.004G301500 in RTx430 or BTx623 to the allele present in ’Rio’ or ’Wray’ genotypes to see if the edited alleles increase WSC.

Considering how few of the genes identified through GWAS have had any true functional annotation, it is critically important that the members of the Poaceae serve as priorities for further gene characterization and validation studies. The recent emergence of alternative pathways unique to certain clades, specifically the C_4_ grasses, demonstrates that homologous comparisons may not be as valuable as perceived. Furthermore, the reliance of homology without genomic loci that have been experimentally implicated through association studies or linkage mapping may not fully capture the genetic determinants of complex genomic interactions unique to the species being studied. Further evaluation of grass genomes and genomics is imperative for the continued increase in their productivity to meet the demand for food, feed, fuel and fiber in the 21^st^ century.
